# Phylogenetic analysis of HPAI H5N1 virus from duck swab specimens in Indonesia

**DOI:** 10.5455/javar.2021.h521

**Published:** 2021-06-28

**Authors:** Dewi Mutisari, Muflihanah Muflihanah, Hendra Wibawa, Ferra Hendrawati, Hamdu Hamjaya Putra, Kartika Priscillia Sulistyo, Ahyar Ahmad, Rizalinda Sjahril, Risna Halim Mubin, Dwi Kesuma Sari, Muhammad Nasrum Massi

**Affiliations:** 1 Master of Biomedical Sciences, Graduate School Hasanuddin University, Makassar, Indonesia; 2 Disease Investigation Center Maros, Directorate General of Livestock and Animal Health Services, Ministry of Agriculture, Maros, Indonesia; 3 Disease Investigation Center Wates, Directorate General of Livestock and Animal Health Services, Ministry of Agriculture, Yogyakarta, Indonesia; 4 Departement of Chemistry, Mathematics and Natural Science Faculty, Hasanuddin University, Makassar, Indonesia; 5 Department of Microbiology, Faculty of Medicine, Hasanuddin University, Makassar, Indonesia; 6 Departement of Internal Medicine, Faculty of Medicine, Hasanuddin University, Makassar, Indonesia; 7 Veterinary Medicine Study Program, Faculty of Medicine, Hasanuddin University, Makassar, Indonesia

**Keywords:** Phylogenetic, HPAI, H5N1, duck, swab

## Abstract

**Objective:**

A phylogenetic study was carried out on the avian influenza virus (AIV) isolated from a disease outbreak in Sidenreng Rappang Regency, South Sulawesi, Indonesia, in 2018.

**Material and Methods:**

Oropharyngeal swabs and organ samples were obtained from ducks that showed clinical symptoms: torticollis, fascial edema, neurological disorders, the corneas appear cloudy, and death occurs less than 1 day after symptoms appear. In this study, isolate A/duck/Sidenreng Rappang/07180110-11/2018 from duck was sequenced and characterized.

**Results:**

It was found that each gene segment of the virus has the highest nucleotide homology to the Indonesian highly pathogenic avian influenza (HPAI) H5N1 clade 2.3.2.1c. Multiple alignments of the sample Hemagglutinin (HA) gene with the avian influenza references virus showed that the pattern of amino acid arrangement in the cleavage site PQRERRRK-RGLF is the characteristic of the HPAI virus. In addition, the HA gene contained Q222 (glutamine) and G224 (glycine), signifying a high affinity to avian receptor binding specificity (SA α2,3 Gal). Furthermore, there was no genetic reassortment of this virus based on the phylogenetic analysis of HA, NA, PB1, PB2, PA, NP, M, and NS genes.

**Conclusion:**

The HPAI H5N1 clade 2.3.2.1c virus was identified in duck farms in South Sulawesi, Indonesia.

## Introduction

Avian influenza virus (AIV) is one of the influenza type A viruses under the Orthomyxoviridae virus family. AIV mainly infects various kinds of birds such as chickens, turkeys, geese, waterfowl, seabirds, and wild birds [1]. However, the AIV can also infect mammals, including humans, such as the case of human bird flu in Hong Kong, where 6 out of the 18 people were infected with AIV and eventually died due to the transmission from birds to humans [2]. AIV consists of eight gene segments that encode PB2, PB1, PA, Hemagglutinin (HA), NP, NA, M (M1 and M2), and NS (NS1 and NS2) proteins [3]. HA is the crucial protein involved in the antigenic properties, pathogenicity, and specificity of viruses to their hosts [4]. The HA protein recognizes the receptors on the surface of the host cell that contains sialic acid [5] and determines the pathogenicity of AIV H5N1 [6].

In December 2003, a disease outbreak in poultry farms was initially detected in Java, Indonesia [7]. The disease was identified as highly pathogenic avian influenza (HPAI) H5N1 clade 2.1 [8]. This virus escalated and resulted as an endemic in Indonesia [9], causing an impact on economic losses [10]. This virus also continues to develop and shows changes such as mutations, changes in pathogenicity, reassortion, or evolution to form the 2.1.1, 2.1.2, 2.1.3, 2.1.3.1, 2.1.3.2, 2.1.3.2a, and 2.1.3.2b clades [11,12]. Then, in 2012, there was a finding of a new AIV clade in Java, called clade 2.3.2.1, in the duck outbreak that had not been previously detected in Indonesia [13]. During its development, this virus clade attacked ducks and other commercial poultry and became predominant in Indonesia [14-16]. Recently, the HPAI H5N1 virus outbreaks are detected in poultry in Indonesia [15-17]. In addition, there are several reports on the HPAI H5NX virus endemic in other regions, including wild and domestic birds in Asia, Africa, and Europe [18].

Sidenreng Rappang Regency is the largest laying hen farms and the third in the total poultry population in South Sulawesi [19]. In 2018, there was an outbreak of AIV in duck farms in Sidenreng Rappang Regency, South Sulawesi, Indonesia. This raised concerns about the virus’ rapid spread to other commercial poultry, including hens, and its effect on human health. Therefore, it is necessary to carry out virus identification and genetic analysis to determine the viral relationship and the possibility of mutations, changes in pathogenicity, and reassortion. In this study, we carried out multiple alignments in cleavage site and receptor-binding site (RBS) using comparisons of total isolates from the Sulawesi island that had never been conducted before. Although some previous studies have reported genetic reassortment of AIVs, no such event has been reported so far in ducks from Sulawesi, Indonesia. This research was conducted to provide molecular information on AIV isolates from ducks in Sidenreng Rappang Regency because genetic characterization in the Sulawesi region, Indonesia, is still limited. The genetic information gained from this study could be a significant reference for implementing the country’s avian influenza (AI) disease control and eradication program.

## Materials and methods

### Ethical approval

The study has been approved by the ethics committee of the Faculty of Medicine, Universitas Hasanuddin, Makassar, Indonesia, with registration number 63/UN4.6.4.5.31/PP36/2021.

### Disease outbreak in South Sulawesi, Indonesia

The Disease Investigation Center (DIC) Maros received a report about a disease outbreak in August 2018. The outbreak began at one of the duck farms and spread to other farms in Sipodeceng Village, Baranti District, Sidenreng Rappang Regency, South Sulawesi Province, Indonesia. Over 3 weeks, 1,350 ducks died with clinical symptoms of torticollis, fascial edema, neurological disorders, cloudy corneas, and death occurred less than a day after the symptoms appear. In addition, some native chickens showed clinical signs and eventually died. Further studies are required to determine the relationship between AI outbreak in ducks and native chicken. However, a positive result of the AI rapid test was carried out by the veterinarian of the Agriculture, Food Security, and Fisheries Department of Sidenreng Rappang Regency.

Oropharyngeal swabs were taken from 20 ducks and 5 chickens with clinical signs, while organs were taken from a duck that had just died. In addition, oropharyngeal swabs pooled from five ducks or native chickens, and organs obtained from a duck were prepared and preserved in Dulbecco’s Modified Eagle Medium tubes and kept at 4°C during transportation to the Virology Laboratory, DIC Maros.

### Isolation and identification of AIV

Isolation of AIV and identification of pool samples were processed following the Office International des Epizooties (OIE) protocol [18]. Of two out of four pools, duck oropharyngeal swab specimens showed the positive AIV H5, while native chicken pool samples and duck organ pool samples were negative. Specimens that showed positive results were stored in a freezer at −80°C. One isolate was selected and then characterized by sequencing based on the highest virus titer in this research.

### Sequencing

The isolate was sent and sequenced at the National Reference Laboratory for AI disease, DIC Wates, Yogyakarta. Sequencing was carried out for whole-genome sequencing (WGS) using the procedure described by Lestari et al. [17] in the previous study. WGS was carried out by multisegment reverse transcription polymerase chain reaction (mRT-PCR) using the next-generation sequencing technique. Simultaneously, mRT-PCR amplified the eight AIV segments with the MBTuni12 and MBTuni13 primers [20] using the SuperScript III One-Step reverse transcription polymerase chain reaction (RT-PCR) Kit (Invitrogen) polymerase chain reaction (PCR) reagent. DNA library preparation and AI genome sequencing were carried out according to the working procedures of the Nextera XT and Miseq Sequencer (Illumina). The sequencing results were compiled, edited, and analyzed using the CLC Genomic Workbench v11.0.1 software. DNA homology analysis was carried out using a search tool in the influenza virus BLAST database at GenBank. The sequence that has been trimmed was then mapped to the selected reference, and then the WGS file was extracted into a FASTA. The FASTA file was then analyzed for nucleotides and amino acids. The nucleotide sequence of each gene segment has been submitted to the genebank with accession numbers MW965492 – MW965499 (Table 1).

### Phylogenetic and molecular analysis

The comparison of each gene’s nucleotide sequence was carried out using phylogenetic and molecular analyzes. They were compared to the reference virus genes AI H5N1 extracted from AIV sequences available in the National Center for Biotechnology Information (NCBI) database. The NCBI isolates selected originally from Sulawesi, Indonesia, from the first one in 2005 to the latest one in 2016. Isolates with sequential isolate codes in the same district or city selected only one isolate per year. We used the Muscle program in the MEGA X software to align the nucleotides of each gene segment and then translate them into amino acids for molecular determinants. The nucleotide sequences of AIVs from the previous Sulawesi isolate were compared to A/Goose/Guangdong/1/96 (ancestor) for molecular analysis of the HA gene. Using MEGA X software, nucleotide sequences were converted into amino acids through an amino acid sequence analysis for molecular determinants. Phylogenetic analysis was also conducted using MEGA X software by neighbor-joining trees with 1,000 bootstrap replications and the Kimura-2 parameter model.

## Results and Discussion

The research identified that the disease outbreak in South Sulawesi, Indonesia, was caused by the AIV subtype H5, based on its clinical symptoms, rapid testing, isolation, and identification. Phylogenetic and molecular analysis was carried out using WGS on an AIV isolate designated as A/duck/Sidenreng Rappang/07180110-11/2018. Zhou et al. [20] explained that sequencing with WGS could be used to amplify various subtypes of the human and animal influenza A virus genomes. Based on the nucleotide BLAST, it was found that A/duck/Sidenreng Rappang/07180110-11/2018 had 97.57%–99.61% nucleotide homology to the Indonesian HPAI H5N1 clade 2.3.2.1c viruses (Table 1). It was lower than previous studies with 99.19%–100% homology to Indonesian clade viruses [17].

HA as the main surface protein of AIV will bind to the host cell receptor [21]. The HA protein is translated as the single HA0 protein. HA must be divided into HA1 and HA2 by a specific site endoprotease proteolytic serine enzyme, usually called the cleavage site area, to activate the virus [22]. The HA protein cleavage site is an indicator of the pathogenicity of the AIV [23]. It is because the HA gene cleavage site will be filled with monobasic or multiple basic amino acids [6]. Therefore, HPAI will find the presence of polybasic amino acid regions. This feature contains five arginines (R) and two lysines (K) [4,23].

According to the multiple alignment analysis of the HA gene, it was discovered that the sample has a similar amino acid pattern of the cleavage site with several references (Table 2). The isolated sample A/duck/Sidenreng Rappang/A07180110-11/2018 showed the PQRERRRK-RGLF pattern. There was a deletion of lysine (K) at position 329. A cleavage site filled with polybasic amino acids indicates that the virus is still classified as HPAI. An identical pattern was also observed in several isolates in 2012, 2013, and 2016. This is consistent with the previous report by Yegani et al. [24], which explains that the PQRERRRK-RG motif on enzymes at proteolytic cleavage is a feature of clade 2.3.2.1c AIVs and belongs to the HPAI category. Previous HPAI H5N1 clade 2.3.2.1c reports on ducks in Indonesia also indicated the presence of polybasic amino acid residues (RERRRKR/G and REKRRKR/G) [15–17,25]. This shows that AIV with the PQRERRRKR/GLF pattern has circulated in various regions in Indonesia, including Sulawesi since 2012, and can still be identified in 2018.

The composition of other comparable amino acids, including isolates in 2009, 2010, and some isolates in 2013, demonstrated a pattern of PQRESRRKKRGLF, which underwent a 325 amino acid substitution position from arginine (R) to serine (S). The amino acid composition has a similar arrangement of amino acids isolated from humans from Indonesia [26]. This amino acid pattern has also been found previously in Indonesia in studies of various poultry in 2006–2011 [27] and live-bird-market samples in 2012–2013 [28]. Meanwhile, the composition of the comparison amino acid isolates from Sulawesi in 2005–2007 showed the same pattern as the A/Goose/ Guangdong isolate (ancestral), namely PQRERRRKKRG. This pattern was found at the beginning of the AI disease outbreak in Indonesia [9,27].

A recent study determined that three amino acid patterns in the HA AIV cleavage sites were developed in Sulawesi, Indonesia, namely PQRERRRKKRGLF, PQRESRRKKRGLF, and PQRERRRK-RGLF. Even though they have different patterns, the three of them still show the polybasic amino acid. Hewajuli and Dharmayanti [29] stated three amino acid patterns in the AIV cleavage site in Indonesia, namely PQRERRRKKRG, PQREGRRKKRG, and PQRESRRKKRG. Wibowo et al. [27] reported five amino acid patterns in the AIV cleavage site in Indonesia, namely PQRERRRKKRG, PQRE-RRKKRG, PQRESRRKKRG, PQRESRRKRRG, and PQRE-RRRKR.

The influenza A virus must recognize a specific receptor (sialic acid) to initiate cell infection [30]. Usually, the HA protein from the human influenza virus will bind to SA-α2.6-Gal, whereas the influenza virus has preferential bonds with SA-α2,3-Gal [30,31]. The HA gene mutation at the RBS can cause changes in receptor binding preferences from avian SA-α2,3-Gal to human SA-α2,6-Gal so that they can be transmitted and enable a pandemic in humans [32]. Amino acids in the RBS of the sample isolates and all reference isolates indicate amino acids Q222 (glutamine) and G224 (glycine) (H3 numbering), as shown in Table 2. This suggests that the AIV subtype H5N1 in this study were not the mutations in the amino acid at RBS, and had a higher affinity for the SA-α2.3 receptor in poultry than human-to-human SA-α2.6 receptors, thus having a lower possibility to spread among human. It can be seen that all isolates from Sulawesi, since the beginning they were found/isolated, still have the same RBS amino acid composition. Previous reports on clade 2.3.2.1c in ducks in Indonesia also showed Q222 (glutamine) and G224 (glycine) [16,17]. Decreased α2.3 binding and increased binding to human α2.6 receptors could also result from a HA sequence mutation, namely the substitution of Q222L and G224S [33,34].

**Figure 1.**
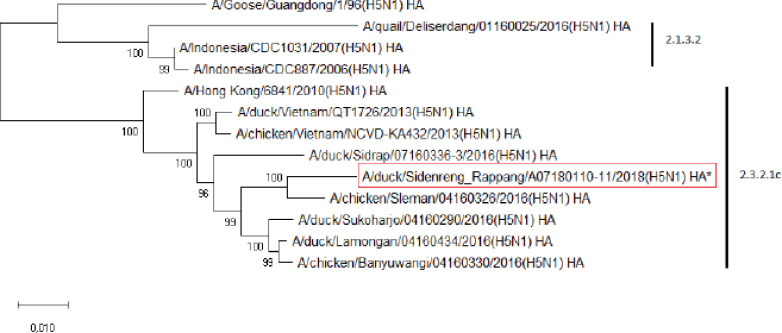
Phylogenetic tree of HA gene segment of HPAI H5N1 virus. An asterisk in the red box represents virus isolate under study.

**Figure 2.**
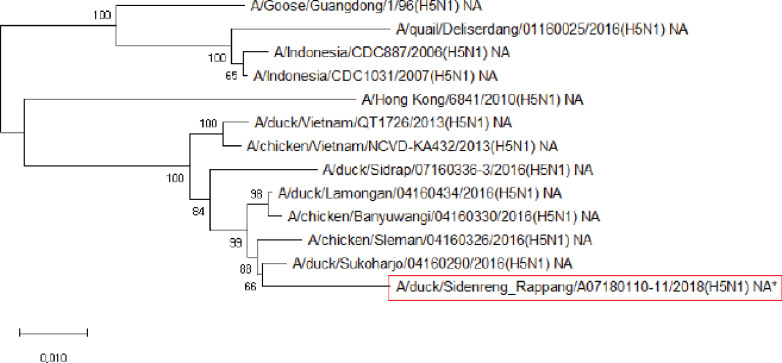
Phylogenetic tree of NA gene segment of HPAI H5N1 virus. An asterisk in the red box represents virus isolate under study.

**Figure 3.**
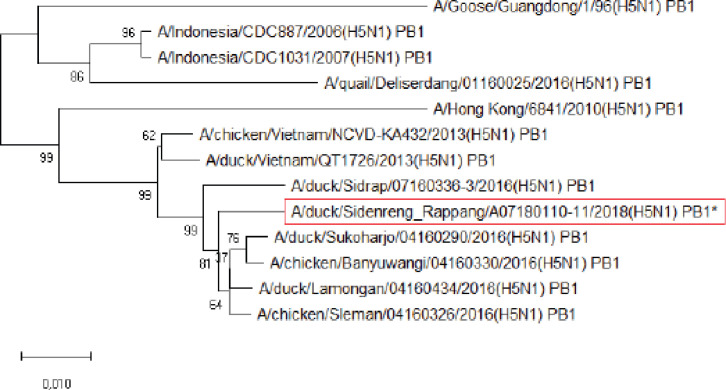
Phylogenetic tree of PB1 gene segment of HPAI H5N1 virus. An asterisk in the red box represents virus isolate under study.

**Figure 4.**
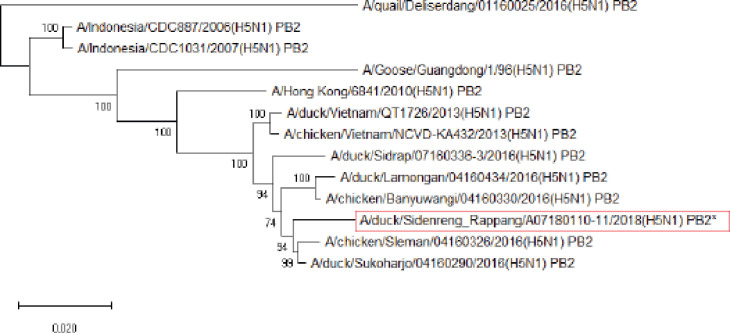
Phylogenetic tree of PB2 gene segment of HPAI H5N1 virus. An asterisk in the red box represents virus isolate under study.

**Figure 5.**
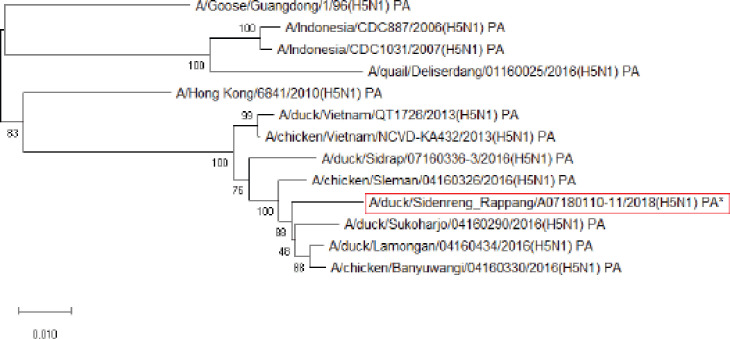
Phylogenetic tree of PA gene segment of HPAI H5N1 virus. An asterisk in the red box represents virus isolate under study.

**Figure 6.**
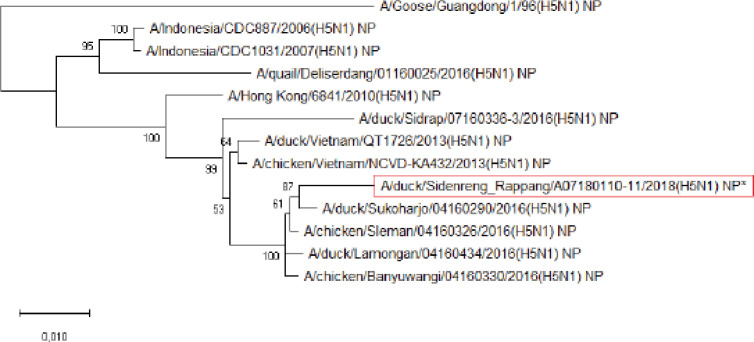
Phylogenetic tree of NP gene segment of HPAI H5N1 virus. An asterisk in the red box represents virus isolate under study.

**Figure 7.**
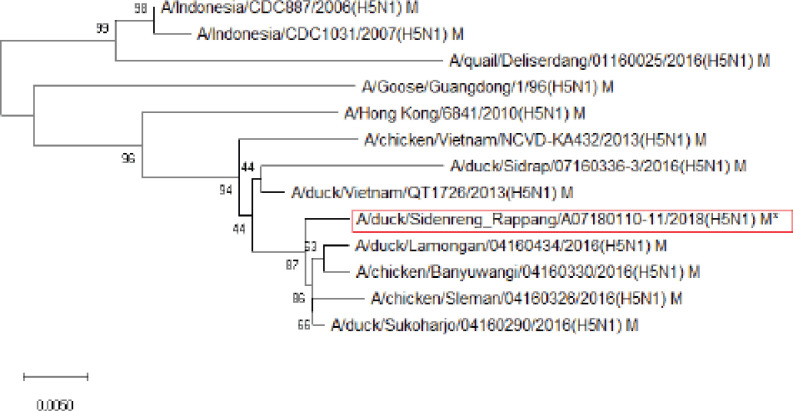
Phylogenetic tree of M gene segment of HPAI H5N1 virus. An asterisk in the red box represents virus isolate under study.

**Figure 8.**
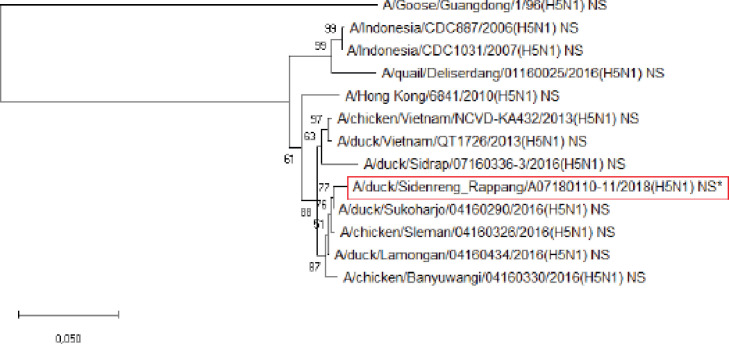
Phylogenetic tree of NS gene segment of HPAI H5N1 virus. An asterisk in the red box represents virus isolate under study.

This study carried out a phylogenetic analysis on 1,776 base pairs of HA gene nucleotides with Indonesian AIV H5N1 isolates and other references from NCBI. The phylogenetic trees of the sample isolate were compared to the reference obtained from a previous study conducted by Wibawa et al. [16], which is a clade 2.3.2.1c that does not show reassortment (Figs. 1-8). Based on the highest nucleotide homology table (Table 1) and the phylogenetic tree (Fig. 1), it is confirmed that our virus is classified as HPAI H5N1 clade 2.3.2.1c. Previous studies showed that outbreaks in duck farms in Sidenreng Rappang were caused by this virus clade . This indicates that this virus is still spreading to duck farms in South Sulawesi, Indonesia.

Further phylogenetic analysis on the eight gene segments exhibited that all genes were similar to the reference isolates, which means there was no genetic reassortment. Wibawa et al. [16] showed three genotypes of the H5N1 clade virus circulating in Indonesia, namely group A, which was the virus group without reassortment, group B, which was the sample group that underwent reassortment on PB2, M, and NS genes, and group C, which underwent reassortment to PB2 genes. Karo-Karo et al. [15], in their research, found that the HA, NA, NP, PA, and PB1 genes were included in two genetic groups in clade 2.3.2.1c, 3 groups of M and NS, four groups of PB2 into four which is one of these group has proximity to Asia’s PB2 LPAI gene. They explained that 13 types of reassortant viruses were mostly detected in backyard chickens among HPAI in Indonesia. Studies on the AI epidemic on ducks in Indonesia recently found two different virus subtypes: HPAI H5N1 and HPAI H9N2. This allows reassortment between the two virus subtypes and leads to new emerging influenza viruses [17]. These findings raise concerns about the potential risk of transmission of the virus among birds as well as threats to human health.

**Table 1.** The highest nucleotide homology of characterized isolates compared to NCBI isolates.

Gene	Sequence length	Accession number	Highest homology virus	% Querycover	% Homology	Clade
PB2	2341	MW965492	(KY614901.1)A/chicken/Sleman/04160326/2016(H5N1)	100	98.12	2.3.2.1c
PB1	2336	MW965493	(KY614902.1)A/chicken/Sleman/04160326/2016(H5N1)	100	98.54	2.3.2.1c
PA	2227	MW965494	(KY614911.1)A/chicken/Banyuwangi/04160330/2016(H5N1)	100	98.11	2.3.2.1c
HA	1776	MW965495	(KY614888.1)A/quail/Sukoharjo/04152003/2015 (H5N1)	100	98.24	2.3.2.1c
NP	1565	MW965496	(KY614905.1)A/chicken/Sleman/0410326/2016(H5N1)	100	98.72	2.3.2.1c
NA	1398	MW965497	(KY614945.1)A/duck/Lamongan/04160434/2016(H5N1)	100	97.57	2.3.2.1c
M	1027	MW965498	(KY614978.1)A/chicken/Sukoharjo/04160454/2016(H5N1)	100	99.61	2.3.2.1c
NS	875	MW965499	(KY614900.1)A/duck/Sukoharjo/04160290/2016(H5N1)	99	99.31	2.3.2.1c

**Table 2.** Analysis of amino acids in the RBS and cleavage site of HA gene of AIV.

No.	Isolate	RBS		Cleavage site												
		222	224	321–333												
1.	A/Goose/Guangdong/1/96	Q	G	P	Q	R	E	R	R	R	K	K	R	G	L	F
2.	A/duck/Parepare/BBVM/2005	Q	G	.	.	.	.	.	.	.	.	.	.	.	.	.
3.	A/chicken/Wajo/BBVM/2005	Q	G	.	.	.	.	.	.	.	.	.	.	.	.	.
4.	A/chicken/SulawesiSelatan/UT2094/2005	Q	G	.	.	.	.	.	.	.	.	.	.	.	.	.
5.	A/chicken/Indonesia/Soppeng/1631-71/2007	Q	G	.	.	.	.	.	.	.	.	.	.	.	.	.
6.	A/chicken/South Sulawesi/M21/2009	Q	G	.	.	.	.	S	.	.	.	.	.	.	.	.
7.	A/chicken/Central Sulawesi/M32/2010	Q	G	.	.	.	.	S	.	.	.	.	.	.	.	.
8.	A/duck/South sulawesi/Sidrap-1021/2012	Q	G	.	.	.	.	.	.	.	.	−	.	.	.	.
9.	A/duck/Westsulawesi/Polman-1018/2012	Q	G	.	.	.	.	.	.	.	.	−	.	.	.	.
10	A/chicken/Minahasa/BBVM 620(2)/2013	Q	G	.	.	.	.	.	.	.	.	−	.	.	.	.
11	A/duck/Konawe/BBVM 19/2013	Q	G	.	.	.	.	.	.	.	.	−	.	.	.	.
12	A/chicken/Barru/BBVM 41-13/2013	Q	G	.	.	.	.	S	.	.	.	.	.	.	.	.
13	A/duck/Sidrap/BBVM 63/2013	Q	G	.	.	.	.	S	.	.	.	.	.	.	.	.
14	A/duck/Sidrap/07160336-3/2016	Q	G	.	.	.	.	.	.	.	.	−	.	.	.	.
15.	A/duck/SidenrengRappang/A07180110-11/2018*	Q	G	.	.	.	.	.	.	.	.	−	.	.	.	.

* Represents the virus isolate under the study, − deletion.

## Conclusion

It can be concluded that the spread of HPAI H5N1 clade 2.3.2.1c was discovered on duck farms in Sidenreng Rappang Regency, South Sulawesi, Indonesia. This has become a growing concern with regard to the future of the rapid spread of the virus with spillover hosts, considering that Sidenreng Rappang Regency is the largest farm area of laying hens in South Sulawesi.

## List of Abbreviations

AIV: avian influenza virus, HPAI: highly pathogenic avian influenza, HA: hemagglutinin, RBS: receptor binding site, WGS: whole-genome sequencing.
